# Discovering Macrophage Functions Using *In Vivo* Optical Imaging Techniques

**DOI:** 10.3389/fimmu.2018.00502

**Published:** 2018-03-15

**Authors:** Yue Li, Tzu-Ming Liu

**Affiliations:** ^1^Faculty of Health Sciences, University of Macau, Macao, China

**Keywords:** bioluminescence imaging, function, intravital microscopy, macrophage, optical imaging, cell tracking

## Abstract

Macrophages are an important component of host defense and inflammation and play a pivotal role in immune regulation, tissue remodeling, and metabolic regulation. Since macrophages are ubiquitous in human bodies and have versatile physiological functions, they are involved in virtually every disease, including cancer, diabetes, multiple sclerosis, and atherosclerosis. Molecular biological and histological methods have provided critical information on macrophage biology. However, many *in vivo* dynamic behaviors of macrophages are poorly understood and yet to be discovered. A better understanding of macrophage functions and dynamics in pathogenesis will open new opportunities for better diagnosis, prognostic assessment, and therapeutic intervention. In this article, we will review the advances in macrophage tracking and analysis with *in vivo* optical imaging in the context of different diseases. Moreover, this review will cover the challenges and solutions for optical imaging techniques during macrophage intravital imaging.

## Introduction

Macrophages, a type of leukocyte, are constitutive cells residing in almost all tissues, from the brain to the skin (1). They are key players in the maintenance of body homeostasis. However, accumulating evidence has indicated that macrophages are involved in a plethora of diseases, such as cancer, infection, diabetes, obesity, atherosclerosis, rheumatoid arthritis, and stroke (1, 2). Better identification and definition of their roles in these contexts will open new opportunities for a better understanding of macrophage functions and dynamics in pathogenesis and homeostasis. However, macrophages have remarkable plasticity and can modify physiology in response to environmental stimuli, which makes it difficult to define a nomenclature fitting all physiological and pathophysiological contexts.

The most commonly used classification method is based on the difference of macrophage activation in the immune response. Classically activated macrophages, or M1 macrophages, respond to bacteria, interferon-γ, lipopolysaccharide (LPS), and tumor necrosis factor (TNF) (3, 4). The second type is defined as alternatively activated macrophages, or M2 macrophages, which respond to interleukin (IL) 4, IL-13, and IL-33 (5). Subsequently, the M1–M2 nomenclature has been rapidly applied to other contexts. For instance, M1 macrophages are involved in atherosclerosis formation by engulfing bulk lipoprotein and in obesity by regulating glucose/lipid metabolism (6, 7), whereas M2 macrophages are involved in wound healing by promoting angiogenesis and tissue remodeling and in cancer progression by assisting tumor development and metastasis (8, 9). However, not all of these macrophages fit the phenotype criteria of the M1–M2 classification. Furthermore, recent discoveries indicate that macrophage functions are not only limited to the previous definition but are also involved in electrical conduction, iron recycling, myofibroblast transformation, synaptic pruning, and HIV persistence (10–14).

As mentioned earlier, macrophage functions are highly educated by the humoral milieu. Traditional phenotype and molecular markers may not be sufficient to determine their dynamic roles and behaviors *in vivo*. Their shapes, migration modes, and adjacent cell compositions in the niche environment need to be considered together to obtain a holistic understanding of versatile macrophage functions. Therefore, a time-course, multiple-labeling, high-spatial resolution *in vivo* observation of macrophage cytomics within a particular microenvironment is required. In this article, we will review the advances in optical imaging techniques for macrophage tracking and analysis *in vivo*, specifically covering examples to show how multiple imaging modalities help us to track and resolve their dynamics *in vivo* under disease contexts. Moreover, this review will cover the challenges and solutions for optical imaging techniques during macrophage intravital imaging. Last, we hope that readers can find appropriate methods and techniques to conduct their own research on macrophage biology from this review.

## Current Methodology for Macrophage Study

Much of our knowledge of the typing and function of macrophages comes from traditional biochemical assays (Table 1). However, the results of these assays are static data averaged over a large number of cells, which cannot reveal the dynamic features of rare cells in this context. To achieve comprehensive understanding, the resolution of biochemical analysis needs to be extended to the single cell level. Single cell sequencing of DNA and RNA is critical for the study of individual cells or few cells in the context of their microenvironment at a higher sensitivity (15). This technique helps to dissect the genetic profiles and signaling pathways that shape the function and behavior of an individual cell (16). Laser microdissection, flow sorting, and microfluidics platforms are three commonly used methods for few cell or single cell isolation (17). The classical case is the uncovering of macrophage function in the electrical conduction of the heart. Hulsmans et al. isolated and purified atrioventricular node macrophages using flow sorting and captured single macrophages by using microfluidic chips. Single cell RNA sequencing analysis showed that atrioventricular node macrophages expressed higher levels of genes involved in electrical conduction, indicating that these resident macrophages may be associated with cardiac conduction (10). Obviously, single cell sequencing has more conclusive advantages in uncovering the function of tissue-specific macrophages compared with traditional biochemical techniques. Although these techniques can capture the evidence at a certain moment of dynamics, they cannot perceive the dynamic functions of macrophages at a specific anatomical location and in their native multicellular microenvironment. This kind of information requires imaging techniques for discovery and visualization.

**Table 1:** Molecular biology and histological techniques for macrophage study *in vitro*.

Markers	*In vitro* techniques	Cell culture	Animal tissue
mRNA	Single cell sequencing	Flow sorting/microfluidics platforms	Laser dissection/flow sorting/microfluidics platforms

	PCR	Cell pellet	Flow sorting

Proteins	Western blotting		

	Flow cytometry	Single cell suspension	Tissue digestion

	Immunofluorescence Immunohistochemistry	Cell smear/cytospin	Tissue section

Secretions	ELISA	Culture supernatant	Blood sample

**Figure 1:**
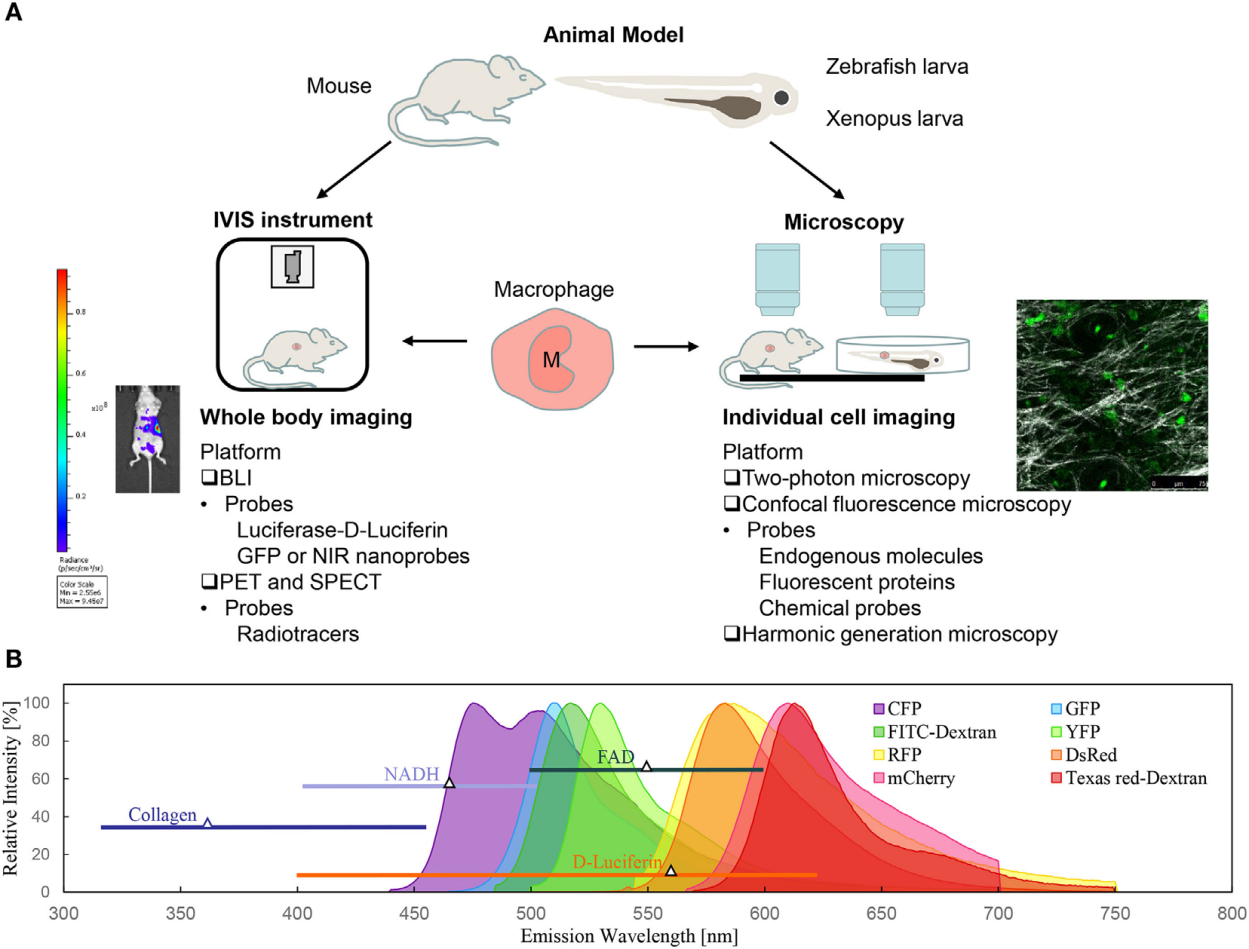
Optical imaging techniques for macrophage tracking *in vivo*. **(A)** Bioluminescence imaging (BLI) and intravital microscopy are the two of most commonly used non-invasive optical techniques for macrophage imaging in animal models. **(B)** Spectra of fluorescent probes for macrophage labeling: intrinsic components (NADH, FAD, and collagen), genetic probes (fluorescent proteins), and commercial chemical probes (fluorescent dye-dextran conjugates).

The dynamic features of immune cells (such as lymphocytes, natural killer cells, and neutrophils) include fast trafficking and high motility, which are prerequisites for these cells to function in immune responses. To track their motion and dynamic functions *in vivo*, much progress in observation techniques has been made in recent years. With the development of smart molecular probes and advanced imaging modalities, optical molecular imaging has provided a feasible approach to visualize single cells or the molecular distribution *in vivo*. Moreover, the physiological dynamics and cytomics can be time-course monitored over days for the same experimental subject. Although various imaging techniques have been applied to track immune cells, such as nuclear imaging (18–20), magnetic resonance imaging (MRI) (21, 22), and photoacoustic imaging (23), their spatial resolution and sensitivity are not sufficient to reveal the underlying mechanisms (24). Here, this review will focus on the optical imaging techniques that can track the dynamic behavior and functional roles of macrophages in different contexts. The approaches can be classified as whole-body imaging and individual cell imaging, with or without labeling (Figure 1A).

## Optical Imaging Techniques

### Bioluminescence Imaging (BLI)

Bioluminescence imaging has become one of the most commonly used non-invasive techniques for whole-body imaging of cell populations in small animal models. BLI is mainly based on the detection of chemiluminescence from the luciferase-mediated oxidation reaction, which has a broad emission spectrum (400–620 nm) and a peak emission wavelength at 560 nm (25). It commonly takes 10–12 min after the injection of luciferin for the emission to initiate, and the light emitted from luciferase slowly decreases after 60 min postinjection. There are three luciferase systems for BLI, including bacterial luciferase, firefly luciferase, and renilla luciferase (26). Luciferin is a kind of light-emitting compound that generates bioluminescence when it encounters luciferase in organisms. The firefly luciferase–luciferin system is the most frequently used contrast combination for cell and animal imaging.

Generally, luciferase-encoding genes are inserted into the promoter region of genes in target cells, and luciferase enzymes expressed in engineered cells then catalyze light emissions during the luciferin oxidation reaction (25). Luciferase-expressing tumor cells have been widely used to monitor tumor growth, detect metastasis, and evaluate responses to therapy. Such reporter systems have also been used for macrophage or bone marrow cell *ex vivo* labeling by utilizing a lentivirus vector that encodes the given reporter gene. The engineered cells are then injected *via* intravenous and orthotopic routes for *in vivo* BLI (27). However, luciferase-labeled primary cells face a technical challenge due to the lower transfection efficiency, as do mouse-derived macrophages (28). Moreover, the short duration of bioluminescence is another problem limiting the popularization of *in vivo* BLI for macrophage imaging.

The alternative solution to achieve long-term and whole-body macrophage tracking is fluorescence imaging. The instrument is equipped with an excitation source of laser, fluorescence filter sets, and a sensitive camera detecting the blue to near-infrared (NIR) wavelength region. The laser can excite the loaded fluorescent dyes/nanoparticles or transgenic-labeled fluorescence proteins in macrophages. Compared with BLI, fluorescence-based imaging has higher signal intensities, and the emission wavelength of labeling can reach the NIR, where deeper imaging depth can be achieved. For instance, green fluorescent protein (GFP) is the first generation engineered protein from *Aequorea victoria*, which exhibits bright green fluorescence (emission peak at 509 nm) when exposed to the light in the blue to ultraviolet range (excitation peak at 488 nm). Both the excitation and emission spectral ranges overlap with the luciferase–luciferin system, so GFP or enhanced GFP (EGFP) can be easily integrated with BLI systems. Compared with luciferase-transfected cells, GFP-transfected cells are more easily prepared but are limited to superficial tissues where both excitation and emission light can penetrate (29). Recently, GFP-transfected macrophages have been successfully established for *in vivo* BLI, which provides a valuable tool to study macrophages under different health and disease contexts (30).

In contrast to fluorescence-based imaging, BLI does not require laser excitation, thus avoiding phototoxicity and background interference. The main disadvantage of *in vivo* BLI is a poor spatial resolution of 1–10 mm and a limited imaging depth of 1–2 cm due to the light scattering of tissues (24, 31). The imaging photons quickly diffuse at depths deeper than 0.5 mm, and the corresponding resolution of images degrades severely at deeper depths. Still, BLI has high sensitivity and high throughput with a 23 cm field of view, allowing for detection with a maximal penetration depth of 3 cm and whole-body imaging (32). Moreover, BLI provides relative quantitative measurements of cell viability and cell function. Due to the requirement of transgenic labeling, the application of BLI is currently limited to preclinical studies.

### Positron Emission Tomography (PET) and Single-Photon Emission Computed Tomography (SPECT)

Positron emission tomography is a nuclear functional imaging technique for whole-body imaging. PET systems can detect pairs of gamma photons emitted by positron-labeled nanomaterials (radiotracer) indirectly. Generally, radiotracers, such as fluorine-18 fluorodeoxyglucose (^18^F-FDG), are composed of biologically active molecules (such as glucose, water, and ammonia) and radionuclides with short half-lives (33). Radionuclides emit a positron when undergoing positron emission decay, and the positron then travels in the tissue until it decelerates to a point where it can interact with an electron. The electron-positron annihilation process produces a pair of gamma rays moving in approximately opposite directions. These paired photons can be intercepted and synchronously detected by a ring of photodetectors. The location of probes can thus be estimated from the arrival time differences of the paired photons, and the three-dimensional (3D) distribution can be reconstructed by a tomography algorithm. Compared with BLI, gamma photons have much less scattering and absorption by the biological tissues. Therefore, PET can sensitively image smaller amounts of cells at a deep imaging depth in large animals and humans. Positron emission tomography-computed tomography (PET-CT) with ^18^F-FDG has become a favored imaging technique in clinical oncology, such as cancer diagnosis, tumor staging, and metastases detection (34). Wonderfully, PET is also used to track inflammation in atherosclerosis in clinical and preclinical practice because atherosclerotic plaques are always beyond the penetration limit of optical imaging techniques (19, 35–37). Several PET nanoparticles for macrophage imaging have been developed in recent years, such as ^18^F-, ^11^C-, ^13^N-, ^89^Zr-, and ^64^Cu-labeled probes (38). PET can visualize cell populations with a spatial resolution of 1 mm to 1 cm, which makes PET more preferable for tissue imaging (24).

Single-photon emission computed tomography is another nuclear imaging technique based on the detection of gamma photons. Without paired photons, SPECT requires collimators and a gamma camera to form images. There are some similarities between SPECT and PET, such as radiotracer use and gamma-photon detection. A radionuclide is attached to a specific ligand, which exclusively binds to a cell population in the targeted tissue. Several radiotracers can be detected at the same time due to the specificity of SPECT tracers. Moreover, SPECT tracers have longer half-lives than those of PET tracers, which makes them preferable for conducting long-term tracking. With the development of nanomaterial-based SPECT probes, such as ^99^mTc-, ^111^In-, and ^125^I-labeled probes, SPECT has been used to track cell population migration and evaluate nanoformulated drug delivery (39). SPECT is also a preferred modality to image atherosclerosis plaques (40). Since the collimators cause large absorption and collimation error, SPECT has poorer resolution and much less sensitivity than PET (24).

### Fluorescence Microscopy

Fluorescence imaging uses endogenous or exogenous fluorescent molecules that are activated by an external light of appropriate wavelength and detects the longer-wavelength, lower-energy light emission at a defined spectral range. Through a conventional microscope framework, fluorescence imaging can be used to observe thinly sliced tissues (5–30 µm thick) or sheets of cultured cells. For thick tissues, fluorescence from different depths will overlap, degrading the image quality. Imaging quality is worse still for *in vivo* observation. Laser scanning intravital microscopy (IVM) is an imaging technique based on advanced laser sources and high axial-resolution microscopy. The two most frequently used imaging modalities of IVM are confocal fluorescence microscopy and two-photon fluorescence microscopy (41).

Confocal fluorescence microscopy enhances the 3D optical resolution and contrast of a micrograph by adding a pinhole placed at the confocal plane in front of the detector to suppress any light coming from out-of-focus planes. Compared with conventional optical microscopy, confocal fluorescence microscopy has the ability to control the depth of field, eliminate out-of-focus background interference, and acquire sectioning images in thick specimens. Limited by the severe scattering and pigment absorption in visible wavelengths, the depth of single-photon excited fluorescence confocal imaging is approximately 80–100 µm (42). To reduce these scattering/absorption effects and achieve deeper imaging depths, two-photon fluorescence microscopy employs NIR femtosecond lasers to excite the same fluorophores (43). It allows imaging of live tissues up to 300–500 µm in depth (42). Since different kinds of immune cells may reside in different depths of layered tissues, such an increase of the imaging depth can broaden the dimension of exploration *in vivo*.

### Fluorescent Probes

Macrophages need to be labeled so that they can be clearly observed under fluorescence imaging. The labeling methods for macrophage imaging have been widely developed with nanoparticle probes and transgenic technology (38, 44), which has resulted in two broad categories of fluorescent probes: genetic probes (fluorescent proteins) and chemical probes (fluorescent molecules) (Figure 1B) (45, 46). Genetic labeling is achieved by transfection of reporter genes that encode fluorescent proteins into the promoter region of genes in target cells, such as *Lysm* and *c-fms* genes for macrophages (44, 47). This is an intrinsic labeling technique, which exerts low disturbance on cell physiologies and allows for long-term cell tracking. Various fluorescent proteins, such as GFP, EGFP, mCherry, yellow fluorescent protein, red fluorescent protein (RFP), and cyan fluorescent protein, have been widely used to label target cells, among which GFP is the most commonly used one for macrophage imaging (48).

Chemical fluorescent molecules are usually divided into endogenous molecules and exogenous fluorescent dyes. Endogenous molecules are metabolic products with optical signatures *in vivo*, such as NADH, FAD, hemoglobin, and collagens. Metabolic cofactors NADH and FAD have been used to identify tumor-associated macrophages (TAMs) in animal cancer models (49). Exogenous fluorescent dyes are widely used for flow cytometry, endoscopy, and intraoperative imaging. The design of exogenous fluorescent dyes for macrophage imaging is mainly based on macrophages’ biological characteristics such as endocytosis activity, specific enzyme secretions, and metabolic preference. Examples are fluorescently labeled dextran or PEG, metal matrix proteinase (MMP) or cathepsin activity-based probes, and folate or mannose-receptor-targeted probes, which have been well reviewed elsewhere (38, 45). IVM combined with the stability of fluorescent proteins or probes enables real-time imaging and long-term tracking of single or multiple macrophages within a tissue *in vivo*. The cellular identity and the functional signaling can thus be visualized in context.

### Harmonic Generation Microscopy

Harmonic generation microscopy exploits second harmonic generation (SHG) and third harmonic generation (THG) as non-linear optical contrasts in laser scanning microscopy. Compared with fluorescence microscopy, harmonic generation microscopy can image cells and tissue structures without labeling, thus providing complementary information on the microenvironment. Various biomaterials, such as collagen, spindle fibers, bones, hemoglobin, melanin, and lipid bodies, have either SHG or THG contrasts in harmonic generation microscopy (50–55). These label-free features have been widely used to visualize the cellular and tissue structures in muscles, the skin, the brain, and the oral mucosa (56–59). Recently, we found that THG microscopy had the capability to image the morphology and granularity of leukocytes, based on which their types were identified and their motion was tracked *in vivo* without any labeling (60, 61). Collagen imaging with SHG microscopy provides a visible background of the collagen structures for tracking macrophage motility and function *in vivo* (62, 63). Importantly, SHG microscopy has the capability to quantify the collagen remodeling (50) and fibrosis (64, 65) in the extracellular matrix (ECM) *in vivo* and can identify the collagen-producing cells (66).

With the advantages of high 3D spatial resolution and time-course tracking capability, multimodal laser scanning IVM is an indispensable platform to obtain a better understanding of macrophage dynamics and functions in pathogenesis.

## Imaging Macrophages in Different Contexts

Based on the IVM platform and advanced molecular probes, the morphodynamics and functional roles of macrophages can be investigated in different contexts. Below, we elucidate how imaging and probes can uncover the macrophage biology in different contexts (Table 2).

**Table 2:** Imaging macrophages in the context of different diseases.

Contexts	Behaviors	Optical platforms	Labeling methods	Models	Reference
Cancer	Origin and recruitment	Bioluminescence imaging (BLI)	Raw 264.7/luciferase	Mouse	(67)
		Intravital microscopy (IVM)	Enhanced green fluorescent protein (EGFP) and EYFP	Mouse	(68)
		IVM	EYFP	Mouse	(69)
	Promoting tumor initiation	IVM	DsRed, EGFP	Zebrafish larva	(70, 71)
		BLI	EGFP	Mouse	(72)
	Guiding angiogenesis	IVM	Red fluorescent protein	Zebrafish larva	(73, 74)
	Assisting tumor invasion	IVM	Texas red dextran, ProSense	Mouse	(75, 76)
	Co-migration with tumor cell	IVM	Green fluorescent protein (GFP), Texas red dextran	Mouse	(77, 78)
	Assisting tumor cell intravasation	IVM	Texas red dextran, enhanced cyan fluorescent protein (ECFP)	Mouse	(79, 80)
	Promoting metastases formation	BLI	Raw 264.7/luciferase	Mouse	(81)

Wound healing	Origin and recruitment	IVM	mCherry	Zebrafish larva	(82)
		IVM	GFP	*Xenopus* larva	(83)
		IVM	ECFP and GFP	Mouse	(84)
	Clearance of microbes and debris	IVM	mCherry and GFP	Zebrafish larva	(85, 86)
	Trapping and engulfing neutrophils	IVM	Dendra2	Zebrafish larva	(87)
		IVM	mCherry	*Xenopus* larva	(83)
	Reducing fibrosis and scarring	IVM	GFP	Mouse	(88)
		IVM	GFP	Mouse	(89)

Obesity	Origin and recruitment	IVM	Acridine orange dye	Mouse	(90)
		IVM	EGFP	Adipose tissue explants	(91)
	Constructing crown-like structure and engulfing lipids	IVM	EGFP	Adipose tissue explants	(92)
	Propagating inflammation	IVM	EGFP	Mouse	(93)
	Metabolizing norepinephrine	IVM	GFP	Mouse	(94)

Atherosclerosis	Origin and cell proliferation	Positron emission tomography-computed tomography (PET-CT)	Fluorine-18 fluorothymidine	Mouse	(37)
	Accumulating in atherosclerotic plaques	PET-CT	^64^Cu radiotracer	Mouse	(19)
		IVM	Near-infrared fluorescence (NIRF) dye	Mouse	(95)
		Intravascular imaging	NIRF dye	Rabbit	(95, 96)

### Cancer

Tumors have a complex microenvironment that consists of cancer cells, lymphatic/blood vessels, stromal cells, immune cells, and secreted proteins, which interact with each other and support the proliferation and metastasis of cancer cells. Among them, TAMs have been shown to play a crucial role in tumor initiation, angiogenesis, invasion and metastasis, immune evasion, and therapeutic resistance, which cover several biological hallmarks of cancer (9, 97–99). During the past decade, optical imaging has successfully helped us unravel the veils masking these complicated relationships between cancer and TAMs.

**Figure 2:**
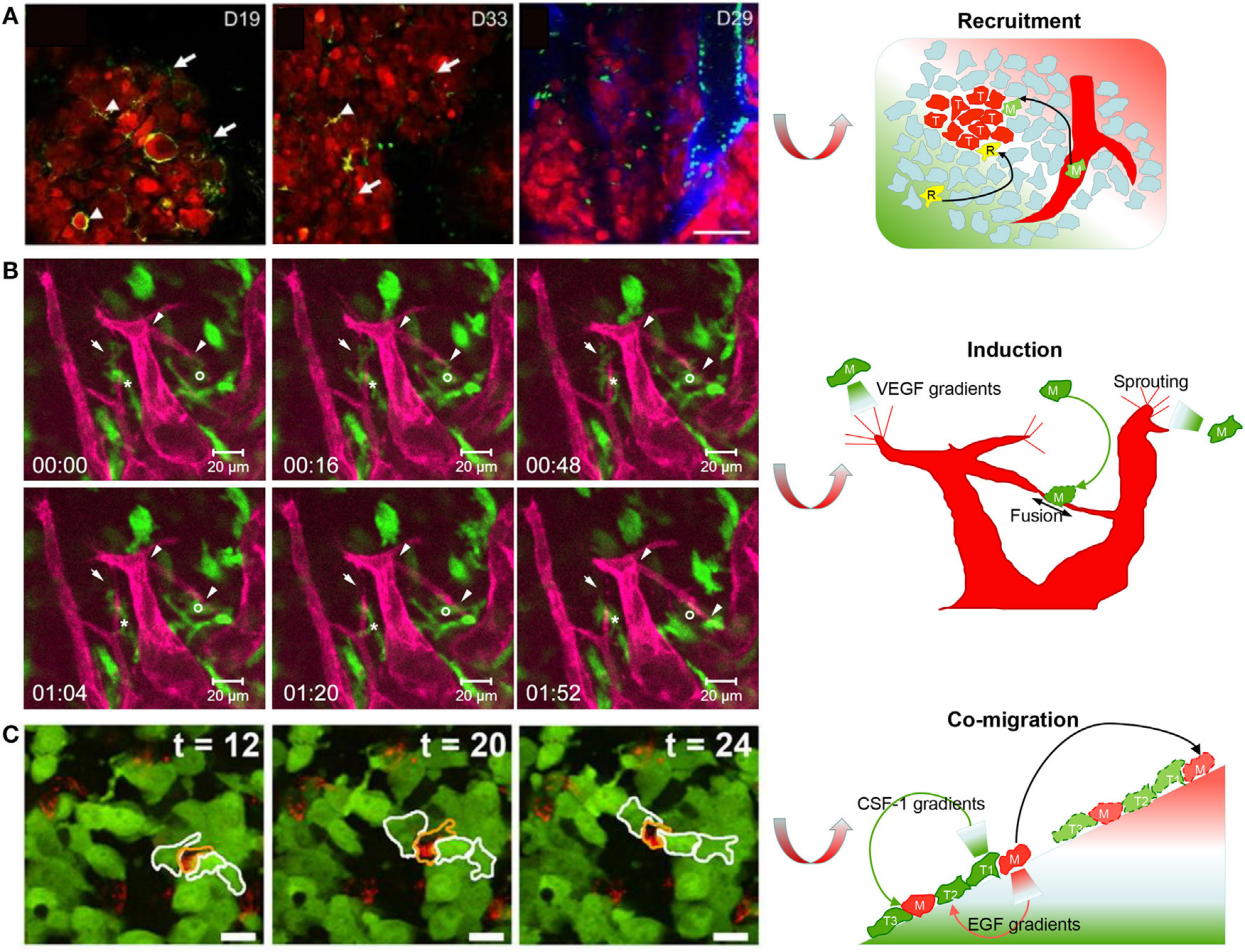
Imaging macrophages in the context of cancer. **(A)** Live imaging on mouse tumor showed that resident CD11c-EYFP^+^ microglia (*R*) were gradually replaced by EGFP^+^/EYFP^+^ monocyte-derived macrophages (*M*) and dendritic cells with glioma development (reproduced under the terms of the CC BY 4.0 license, Copyright© 2016 Springer Nature.) (68). **(B)** Live imaging in mouse cornea showed that one enhanced green fluorescent protein (EGFP)-labeled macrophage (*M*)-guided mCherry-labeled endothelial branch elongation by promoting endothelial tip cell sprouting [reproduced under the terms of the CC BY 4.0 license, Copyright© 2015 Hsu et al. (73)]. **(C)** Texas red dextran-labeled macrophages (*M*) co-migrated with EGFP-expressing tumor cells (*T*) as part of a migratory stream according to live imaging *in vivo* [reproduced with permission from Ref. (78), Copyright© 2011 the Company of Biologists Ltd.].

Since TAMs support the tumor biology in many ways, a better understanding of their origins will help to limit cancer development. There are two main origins for tissue-resident macrophages under homeostasis. One is embryonic progenitor-derived resident macrophages with self-renewal ability, and the other is bone marrow-derived monocytes in peripheral blood (100, 101). However, which of these is the major origin of TAM precursors remains speculative and may depend on the type of tumor. Ricard et al. validated that resident microglia (resident macrophages in the brain) were recruited to a glioblastoma in mice. A spheroid of DsRed tagged GL261 glioma cells was injected *via* orthotopic routes into an immunocompetent LysM-EGFP//CD11c-EYFP double-transgenic mouse. Two-photon fluorescence microscopy showed that the glioma microenvironment was dominated by invading CD11c-EYFP^+^ microglia from adjacent tissues during the early stages of tumor development, followed by bulk LysM-EGFP^+^ macrophages and dendritic cells derived from circulating monocytes (Figure 2A). At the latest stages of glioma progression, the density of microglia decreased by half, whereas the density of monocyte-derived macrophages and dendritic cells strongly increased. This study successfully elucidated the infiltration dynamics of tissue-resident macrophages and monocyte-derived macrophages in a glioblastoma microenvironment (68). By contrast, Movahedi et al. found that TAMs surrounding mammary tumors, implanted in the mammary fat pad, were almost exclusively derived from Ly6C^hi^ monocytes using an *in vivo* monocyte labeling technique (102). Choi et al. used *in vivo* BLI to visualize the recruitment process of circulating macrophages (Raw 264.7 cells) toward mouse colon tumors. The Raw 264.7 cells expressing enhanced firefly luciferase (effluc) migrated into the tumor periphery after intravenous injection in live mice. These studies validated the idea that modified monocytes, or even transfused macrophages, may be candidate carriers to deliver cancer drugs to tumors (67). However, the pharmacokinetics will depend on the tissue location and the types of cancer.

Recently, Swirski et al. showed that the spleen was another reservoir site for blood monocytes. With the help of IVM, they found that GFP-labeled monocytes departed from the spleen into the blood circulation, flowing to the ischemic acute injury heart in Cx3cr1^gfp/+^ mice (103). Coincidentally, new evidence has revealed that spleen-derived monocytes are also an origin of TAMs for lung cancer. Cortez-Retamozo et al. revalidated the origins of TAMs in a conditional genetic mouse model of lung cancer. They found that the spleen also produced Ly6C^hi^ monocytic cells locally during cancer progression. IVM indicated that large numbers of EYFP^+^ granulocyte/macrophage progenitors (GMPs) colonized the spleen 5 days after fluorescently tagged GMPs injection. High numbers of TAMs were derived from splenic GMPs after tumor initiation. Spleen resection had a significant inhibition on TAM recruitment in lung tumors (69). Given these findings, TAMs are mainly derived from bone marrow-derived monocytes and splenic monocytes, which may provide new opportunities to treat cancer by targeting the progenitor cells of TAMs.

Regarding tumor initiation, there is a growing hypothesis that chronic inflammation is the root cause for various cancers. During initiation phases, macrophages create an inflammatory and mutagenic environment by generating reactive oxygen and nitrogen species which can cause gene mutations in neighboring epithelial cells (104). Feng et al. for the first time revealed the earliest interactions between the host immune environment and oncogene-transformed cells. Live imaging using a zebrafish larvae model showed that H_2_O_2_ was a regulator for initiating the host inflammatory response to oncogene-transformed cells, and blocking the host inflammatory response to oncogene-transformed cells significantly reduced the numbers of transformed cell clones (70). Weber et al. used a transgenic mouse model of wound-induced skin tumorigenesis to investigate the role of macrophages during the earliest stages of tumor formation. Sublethally irradiated mice were transplanted with bone marrow cells isolated from CD11b-DTR donor mice in which monocytes/macrophages were labeled with a DTR-EGFP fusion protein. BLI showed that the abundance of macrophages in wounds was predictive of tumor formation. The number of macrophages in wounds reached a peak 5 days after wounding, with an elevated tumor risk. The authors concluded that macrophages activated during wound healing played a crucial role in early inflammation-mediated skin tumorigenesis, more specifically, that macrophages promoted epidermal cell proliferation by sustained secretion of arginase-1 (72). Hamilton et al. also confirmed that microglia played a prominent role in human glioblastoma formation in the zebrafish brain using live imaging. Three kinds of human glioblastoma cells expressing mCherry were transplanted into transgenic zebrafish in which the microglia expressed EGFP. Mutant-lacking microglia confirmed that macrophages promoted tumor cell survival and invasiveness in zebrafish (71). These cases demonstrate that TAMs are accomplices to tumor initiation.

During progression phases, TAMs can switch on angiogenic processes to resolve hypoxic stress by secreting angiogenic growth factors and cytokines, such as VEGF, IL-8, FGF, and PIGF (105). Angiogenesis is a two-step process that begins with vessel sprouting followed by vessel fusion. Hsu et al. examined the dynamic cell–cell contact between macrophages and sprouting vessels in Csf1r-EGFP^+/tg^ and Flk1-myr-mCherry^+/tg^ double-transgenic mice using IVM. Live imaging indicated that EGFP^+^ macrophages directly facilitated vessel anastomosis and vessel sprouting by bridging endothelial tip cells (Figure 2B) (73). Moreover, Fantin et al. also showed the spatiotemporal relationship between macrophages and blood vessels during angiogenesis in zebrafish. Live imaging showed that RFP-labeled macrophages guided GFP-labeled tip cell sprouting and neighboring vascular fusion by creating VEGF chemotactic gradients. Moreover, the authors verified that there were remarkable similarities between the proangiogenic tissue macrophages during embryonic development and TAMs that promoted angiogenesis in cancer (74). These findings present the possibility that TAMs may be a potential target for antiangiogenic therapies (106).

Pioneering studies showed that TAMs and dendritic cells were able to present antigens to cytotoxic T cells and macrophages, which were modified to kill tumor cells (107). For example, Moalli et al. revealed that the capture of tumor-derived antigen (TDA) by macrophages induced a humoral immune response in mice using intravital imaging (108). Melanoma cells B16.F10-tdTom expressing the fusion protein (as surrogate TDA) tdTomato RFP and coexpressing luciferase were injected into mice with macrophages expressing GFP. tdTom^+^ TDA was engulfed by GFP-labeled macrophages located in the subcapsular sinus. Moreover, depletion of macrophages or genetic ablation of B cells resulted in dramatically reduced TDA capture in tumor-draining lymph nodes, suggesting that TAMs played an essential role in antigen presentation to lymphocytes (108). Furthermore, Yang et al. presented that PC-3-RFP prostate cancer cells were engulfed and digested by GFP-labeled TAMs in a transgenic GFP nude mouse. Moreover, many GFP-expressing dendritic cells directly contacted B16F10-RFP melanoma cells with their dendrites 3 weeks after tumor implantation (109). However, TAMs do not suppress tumor development in substantial types of tumors. Recent discoveries indicate that TAMs block tumor immunosuppression by controlling circulating monocyte recruitment and polarization and by inhibiting cytotoxic T cell responses (110–112).

Tumor-associated macrophages are involved in almost all processes related to tumor cell invasion and metastasis. Migration toward blood vessels and tissue invasion are prerequisites for distant tumor metastasis. TAMs facilitate tumor cell migration by remodeling the ECM during tumor development, especially at sites of tumor invasion (63). The ECM comprises a 3D supramolecular network of polysaccharides and proteins, including collagen, glycoproteins, and proteoglycans. TAMs remodel the ECM (specifically collagen fibers) by using MMPs and the cathepsin enzyme family. Madsen et al. showed that macrophages were involved in ECM degradation. Fluorescently labeled collagen was extensively engulfed by Texas red dextran-labeled macrophages in an MMP-dependent pathway and was subsequently routed to lysosomes for complete degradation (75). Moreover, Onda et al. showed that TAMs initiated tissue remodeling at the tumor periphery by secreting cathepsin. ProSense is a commercial protease activity-based fluorescent probe. Cathepsin labeled by ProSense was specifically localized in macrophages. ProSense signals were observed primarily in stromal tissue at the tumor periphery, where tissue-remodeling activity was high to facilitate tumor cell invasion (76).

Tumor-associated macrophages assist tumor cell invasion by creating chemotactic gradients. Using live imaging, Wyckoff et al. provided the first direct evidence for a synergistic interaction between TAMs and tumor cells during cell migration *in vivo*. In this study, microneedles filled with matrigel and containing growth factors were used to mimic a chemotactic source. Intravital imaging in tumor-bearing mice showed that dextran-labeled TAMs expressing CSF-1 receptor and GFP-labeled tumor cells expressing EGF receptor co-migrated toward microneedles containing either EGF or CSF-1. This work indicated that maintaining an EGF and CSF-1 paracrine loop between tumor cells and TAMs was required for the migration of breast tumor cells to the surrounding tissue and blood vessels (77). By using *in vivo* live imaging, Roussos et al. directly showed that Texas red dextran-labeled TAMs co-migrated with EGFP-expressing tumor cells as part of a migratory stream (Figure 2C). Moreover, macrophage depletion using clodronate liposomes verified that the tumor cell migratory streaming required the presence of TAMs *in vivo*. This work also confirmed the involvement of the EGF and CSF-1 paracrine loop in tumor cell migratory streaming by using erlotinib to block the EGFR on tumor cells or by using CSF1R antibodies to block the CSF1R on macrophages (78).

Intravasation is the crucial step for tumor hematogenous metastasis. Wyckoff et al. used intravital multiphoton microscopy to validate that perivascular TAMs contributed to tumor intravasation in live animals for the first time. The motility of GFP-labeled tumor cells occurred most frequently in association with Texas red dextran-labeled perivascular TAMs (79). Furthermore, the tripartite arrangement of an invasive tumor cell, a macrophage, and an endothelial cell form a tumor microenvironment of metastasis (TMEM), which can assist the intravasation of disseminated tumor cells. TMEM density was greater in systemic metastatic breast cancer patients than in patients with only primary cancer, indicating that TMEM was associated with hematogenous metastasis (113). Harney et al. revealed how TAMs in TMEM assist tumor cell intravasation by using real-time imaging. Time-lapse IVM showed that GFP-expressing tumor cells and ECFP-expressing macrophages streamed toward tetramethylrhodamine dextran-labeled vascular branch points where non-migratory TMEM was predominantly located. Once tumor cells and macrophages arrived at vessels, macrophages in TMEM secreted VEGFA to regulate vascular permeability transiently and assisted tumor cell intravasation. Depletion of macrophages or VEGFA confirmed that TMEM-associated macrophages and VEGFA signaling from TAMs were essential for vascular permeability modification and tumor cell intravasation (80).

After intravasation, the next steps for distant metastasis include cell arrest, extravasation, and micrometastases formation. It has been reported that there is a distinct population of macrophages recruited to the distant metastatic sites, which promotes the extravasation, seeding and persistent growth of tumor cells (114). Furthermore, the recruitment of metastasis-associated macrophages was dependent on CCL2–CCR2 signaling. CCR2 (the receptor for chemokine CCL2) is expressed on blood monocytes, while CCL2 is synthesized and secreted by both tumor cells and stroma cells. Inhibition of CCL2–CCR2 signaling significantly reduced the number of recruited monocytes and inhibited metastatic seeding *in vivo* (115). Li et al. used BLI to show that macrophages have a key role in the initiation steps of lung metastasis. Raw 264.7 cells and anaplastic thyroid cancer CAL-62 cells were transfected with effluc for *in vivo* BLI. Macrophage depletion confirmed that macrophages assisted tumor cell seeding *in vivo* after CAL-62/effluc cell injection (81). These findings indicate that targeting TAMs in primary tumor or macrophages in metastatic organs may be a method to block cancer metastasis.

In summary, macrophages promote primary and secondary cancer progression and metastasis. Moreover, increasing evidence has shown that macrophages regulate tumor responses to anticancer therapies, such as by promoting tumor regrowth, angiogenesis, and metastasis after treatment cessation (97, 116). Therefore, recognizing the roles of TAMs not only enriches the understanding of cancer biology but also inspires the development of new cancer therapies.

### Chronic Wounds and Scarring

The inflammatory response plays a critical role in the healing of wounds after injury. Without proper treatment, fibrosis and scars may form. In diabetic conditions, dysfunction of healing will cause problematic wounds threatening the patient’s health (117). Regardless of wound type, macrophages are prominent cell types in the microenvironment and have different functions at different stages of healing dynamics (118). They are pivotal factors in wound immunoediting (119, 120).

Platelets are the first cell population to be recruited at sites of injury, preventing further blood loss from damaged vessels. Next, neutrophils arrive at the wounds, kill microbes, and clear cellular debris (118). Kim et al. used *in vivo* BLI to examine the kinetics of neutrophil infiltration during wound healing. EGFP-tagged neutrophils colonized the wound as soon as 4 h after the skin injury. The number of neutrophils rapidly increased fivefold from 4 to 18 h and reached a peak between days 1 and 2, before sharply decreasing at day 5 after injury. Moreover, a high rate of EGFP^+^ neutrophil turnover was observed within 6 h after injury (121). This means that continuous elimination and replenishment of neutrophils occur in the wound microenvironment.

**Figure 3:**
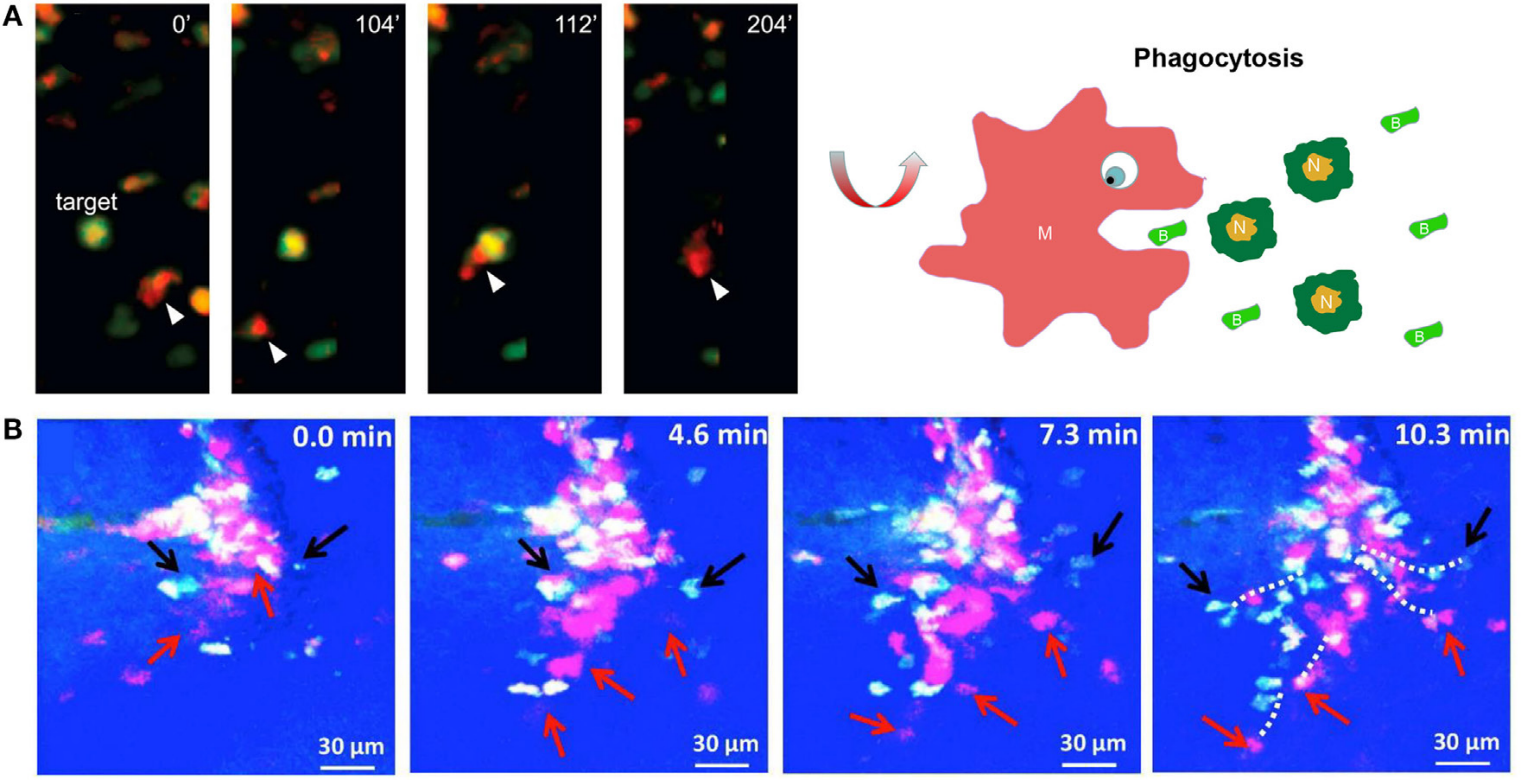
Imaging macrophages in the context of wound healing. **(A)** Intravital imaging showed that mCherry-labeled macrophages engulfed green fluorescent protein-expressing necrotic/apoptotic neutrophils (*N*) at the wound site in transgenic *Xenopus* larva [reproduced under the terms of the CC BY 4.0 license, Copyright© 2015 Paredes et al. (83)]. **(B)** Time-lapse imaging showed that ECFP^+^ monocytes and PE-labeled neutrophils concomitantly infiltrated the wound bed through microhemorrhages in the skin wound of mice [reproduced under the terms of the CC BY 4.0 license, Copyright© 2014 Rodero et al.] (84).

Regarding the elimination mechanism, the current paradigm indicates that infiltrating neutrophils undergo apoptosis at the site of inflammation, where they are subsequently recognized and ingested by neighboring macrophages (122, 123). The triggering of neutrophil apoptosis may originate from the proapoptosis cues in the inflammatory microenvironment of wounds. Neutrophils integrate these signals through surface receptors such as β2 integrins, and common downstream mechanisms lead to the promotion of their apoptosis (124). Increasingly, more findings indicate that macrophages are involved in local neutrophil apoptosis and resolution at wounds. Wound macrophages produce various cytokines, such as TNF-α, to induce neutrophil apoptosis in wounds through a constitutive effector mechanism requiring both intercellular binding through integrin–ligand interactions and membrane-bound TNF-α (125). Such apoptosis mechanism requires a cell-to-cell contact and can be validated through *in vivo* microscopy. Another possible elimination process of neutrophils is the direct engulfment by macrophages. Tauzin et al. used real-time imaging to record the process of trapping and phagocytosis of a DsRed-labeled neutrophil by a Dendra2-labeled macrophage in zebrafish (87). Moreover, Paredes et al. provided direct evidence that mCherry-labeled macrophages engulfed GFP-expressing necrotic/apoptotic granulocytes at the wound site in double-transgenic *Xenopus* larva (Figure 3A) (83).

However, Yoo et al. proposed another mechanism of neutrophil resolution. They demonstrated that Dendra2 (a photoswitchable fluorescent protein)-expressing neutrophils emerged from blood vessels to wounds, and migrated back-and-forth between the injured tissues and vessels. Subsequently, wound-activated neutrophils dispersed from injured tissues into the rest of body as wound healing proceeded. The authors speculated that the underlying mechanisms of neutrophil reverse migration may be implicated in the pathogenesis of systemic immunity (126). Woodfin et al. developed an *in vivo* four-dimensional imaging platform for analysis of leukocyte transendothelial cell migration in mice. Real-time confocal imaging showed that inflammation following ischemia–reperfusion injury triggered multiple forms of transendothelial cell migration, such as extravasation and intravasation. Reduced expression of junctional adhesion molecule-C resulted in neutrophil reverse transendothelial cell migration *in vivo*. They also found that retrograde neutrophils contributed to dissemination of systemic inflammation (127). Subsequently, Colom et al. found that the leukotriene B4-neutrophil elastase axis was a promoter of neutrophil reverse transendothelial cell migration in mice (128). Recently, Tauzin et al. used live imaging to describe how macrophages resolve inflammation by inducing neutrophil reverse migration at wounds in zebrafish. They found that once DsRed-labeled neutrophils arrived at wounds, Dendra2-labeled macrophages often contacted neutrophils and shepherded neutrophils away from wounds through redox and Src family kinase signaling (87). The reverse migration of neutrophils is still a controversial phenomenon. More details can be found in Nourshargh’s review (129). Studies using IVM in the past 10 years have provided undisputed evidence for the ability of neutrophils to exhibit reverse migration behaviors in mice and zebrafish under inflammation and injury contexts (87, 126–128). Macrophages may play a role in this process. A better understanding of the characteristics and pathophysiological relevance of neutrophil reverse migration events may provide a novel strategy to monitor and control disease progression such as cytokine storm in acute lung injury.

Current consensus suggests that macrophages arrive at wounds a little later than neutrophils (130). Gray et al. used intravital imaging to verify that macrophages were recruited to the site of injury more slowly than neutrophils in zebrafish. Importantly, this work developed a novel transgenic zebrafish for IVM, in which neutrophils and macrophages were simultaneously labeled by different fluorescent proteins. Compared with GFP-expressing neutrophils, mCherry-expressing macrophages migrated more slowly to a new wound. Moreover, the number density of GFP-expressing neutrophils in injured tissues reached a peak at approximately 6 h postinjury before injury resolution, while mCherry-tagged macrophage recruitment increased until at least 48 h postinjury (82).

Regarding origin, wound macrophages originate primarily from circulating blood monocytes (120). Paredes et al. investigated the morphology and migratory behavior of labeled myeloid cell populations during tissue repair and regeneration following injury in *Xenopus* larvae. After mechanical or UV injury, GFP-expressing myeloid cells rolled along the endothelium of vessels, were arrested at the injured area, and were then extravasated across the endothelium to wounded tissues (83). Recent evidence showed that resident macrophages are another origin for wound macrophages. Rodero et al. used *in vivo* imaging to reveal the movement of immune cells recruited to skin wounds. This system had the capability to distinguish monocytes (ECFP^+^GFP^+^) from neutrophil (ECFP^low^GFP^−^) and natural killer cell (GFP^+^) subsets based on the expression levels of ECFP and GFP. Within hours after skin injury, ECFP^+^GFP^+^ monocytes and ECFP^low^GFP^−^ neutrophils infiltrated the wound bed concurrently. Monocytes migrated to the injured tissues from the bloodstream through microhemorrhages rather than through the commonly known transendothelial migration (Figure 3B) (84). These findings were not consistent with previous studies, and the differences may result from the wound model and injury pattern. Moreover, live imaging showed that few ECFP^+^GFP^+^ cells (most likely tissue-resident macrophages) immediately appeared in the deep skin wounds after the injury. The ECFP^+^GFP^+^ cell number remained constant for the first 3 h after injury and then rapidly increased by 4 h, and the density of these cells dropped thereafter. This study also showed that both resident macrophages and blood monocytes could be an origin for wound macrophages (84).

Regardless of whether macrophages arrive at wounds earlier or later, they are involved in almost all phases of wound healing. In the early phases, resident and recruited macrophages are classically activated. Classically activated macrophages, as phagocytes, carry out phagocytosis of all microbes, matrix, and cell debris of platelets and secrete pro-inflammatory mediators and chemokines to recruit more circulating monocytes (130, 131). Clearance of microbes through phagocytosis is an essential step to avoid infection in wounds. Macrophages and neutrophils are the main phagocytes engulfing bacteria during wound healing. Mostowy et al. used live imaging to study the interactions between bacteria and phagocytes. The results showed that GFP-labeled Shigella was rapidly engulfed by mCherry-expressing macrophages and neutrophils in zebrafish larva (85). Cellular debris clearance by phagocytic cells is a crucial step in tissue repair and wound healing. Rasmussen et al. revealed that phagocytosis of neuronal debris (red) created by injury was performed by GFP-expressing vertebrate epidermal cells in transgenic zebrafish (86). This work indicated that vertebrate epidermal cells may be one kind of broad-specificity phagocyte functioning as a macrophage. In the later phases of wound repair, macrophages produce various cytokines to induce neutrophil apoptosis and thereafter engulf apoptotic neutrophils for local inflammation resolution in wounds (125).

Importantly, it is essential for macrophages to switch from the classically activated phenotype (pro-inflammatory) to the wound-healing phenotype (anti-inflammatory) during wound healing (119). Accumulating evidence has demonstrated that decreased content and dysregulation of macrophages were related to the dysfunctional healing of diabetic wounds (132–134). Kim et al. used BLI to demonstrate that increased trafficking of the pro-inflammatory macrophages to wounds resulted in persistent inflammation and impaired wound healing. BLI showed that prolonged exposure to epinephrine resulted in persistent recruitment of EGFP^+^ neutrophils to wounds, which impaired wound repair. However, pro-inflammatory macrophage-derived IL-6 was the critical cytokine for neutrophil recruitment. This study showed that stress-induced hormones impaired wound healing by altering the inflammatory response in wounds (135).

Accumulating observations have suggested that wound and cancer tissues share common cellular and molecular activities. In a classic publication, Dvorak formulated his famous hypothesis that tumors were wounds that did not heal (136). This hypothesis has been strongly supported by experimental studies. In the later phases of wound repair, wound-healing macrophages secrete growth factors (such as VEGFA, VEGFC, and MMPs) that stimulate fibroblast proliferation, lymphangiogenesis and angiogenesis (8, 137). In the very late phases, as the wound resolves, macrophages promote capillary regression and collagen remodeling to reduce fibrosis and scarring (119). This is a subtype of macrophage with anti-fibrotic and antiangiogenic activity, which is different from M2 macrophages or wound-healing macrophages. Dreymueller et al. conducted a wound-healing assay to evaluate the effect of embryonic stem cell-derived M2 macrophages on mouse tail wounds. They found that prolonged exposure of PKH67 green-tagged M2 macrophages delayed wound healing and promoted more scar formation in deep skin wounds. This study indicated that different subtypes or lineages of M2 macrophages may not promote healing (88).

Scar formation is the result of abnormal healing and may cause symptoms such as pain, cirrhosis, or pulmonary fibrosis. For example, wound-derived corneal scarring is one of the leading causes of blindness. MMP12, a metalloelastase with elastase activity, has been shown to regulate fibrosis during wound healing under various injury models (138). Chan et al. used live imaging to reveal that MMP12 has a protective effect on corneal scarring during wound repair. Intravital imaging demonstrated that MMP12 deficiency increased the accumulation of EGFP-labeled myeloid cells (macrophages) in Mmp12^−/−^ mouse corneal wounds. Moreover, macrophage-derived MMP12 blunted the corneal myofibroblast transformation and angiogenic response to avoid fibrosis during wound repair (89). Myofibroblast transformation is the main contributor to scar formation during wound healing and is the main cause of organ fibrosis during chronic or acute injury, such as liver fibrosis, renal fibrosis, and lung fibrosis (139). However, the developmental origin of myofibroblasts remains controversial. There are many possible origins of myofibroblasts, such as smooth muscle cells, epithelial cells, resident progenitor cells, and fibroblastic cells (140, 141). Recent discoveries based on cell lineage tracing studies indicate that macrophages can transdifferentiate into myofibroblasts during human and experimental renal fibrosis (12, 142, 143). However, the macrophage-to-myofibroblast transition (MMT) is still an area under investigation. The critical step that marks the MMT is the collagen production from macrophages. Therefore, intravital two-photon fluorescence and SHG microscopies can monitor this critical process and may provide more visual evidence of the MMT *in vivo* in the future.

Local inflammation resolution is a crucial step for the return to tissue homeostasis. Regarding the fate of wound macrophages, they may be eliminated by local apoptosis, by emigrating to draining lymph nodes and the spleen, or through a combination of these pathways (144–146). As mentioned, landmark evidence has shown that macrophages are key regulators in wound healing (119, 120). Further investigations may provide a potential therapeutic approach for avoiding scarring in wound healing or for impaired wound healing in diabetic patients.

### Obesity

It is generally agreed that obesity brings chronic inflammation of adipose tissues, and macrophages are key partners in this process (147). Accumulating evidence has demonstrated that increased macrophage infiltration in adipose tissue is positively related to obesity development (7, 148, 149). Macrophages infiltrate adipose tissues and accumulate around dead adipocytes, which form crown-like structures (CLSs) (150). A CLS is the primary site for the proliferation of adipocyte precursors and the activation of macrophages (151). Therefore, CLS formation is an inflammatory hallmark of adipose tissue (91).

Under homeostasis, resident adipose tissue macrophages (ATMs) mainly arise from bone marrow precursors and blood monocytes (152, 153). Surprisingly, little is known about the origins of ATMs in obese adipose tissue. To visualize the dynamics of macrophage recruitment *in vivo*, Nishimura et al. used FITC-dextran and acridine orange dye to label vessels and leukocyte nuclei, respectively, in genetically obese mice. Erythrocyte, leukocyte, and platelet cells were identified according to the cell size. Live imaging showed that leukocyte–endothelial cell–platelet interaction, a hallmark of inflammation, increased in the microcirculation of obese adipose tissue. Leukocyte rolling, firm adhesion to endothelial cells, and extravasation were observed. These findings indicated that blood monocytes were the sources of ATMs in obese adipose tissue, and ATMs contributed to the activation of local inflammation in obese mice (90). Moreover, there is an interesting hypothesis that local proliferation is another origin of ATMs in obese adipose tissue. Haase et al. used *ex vivo* live imaging of adipose tissue explants from CSF1R-EGFP mice to verify this hypothesis. Live imaging showed that EGFP-tagged macrophages preferentially increased near the CLS; afterward, some macrophages emigrated out of the CLS to become resident in the interstitium. These data confirmed that the CLS was a niche for macrophage proliferation and a new source of ATMs in obese adipose tissue (91).

**Figure 4:**
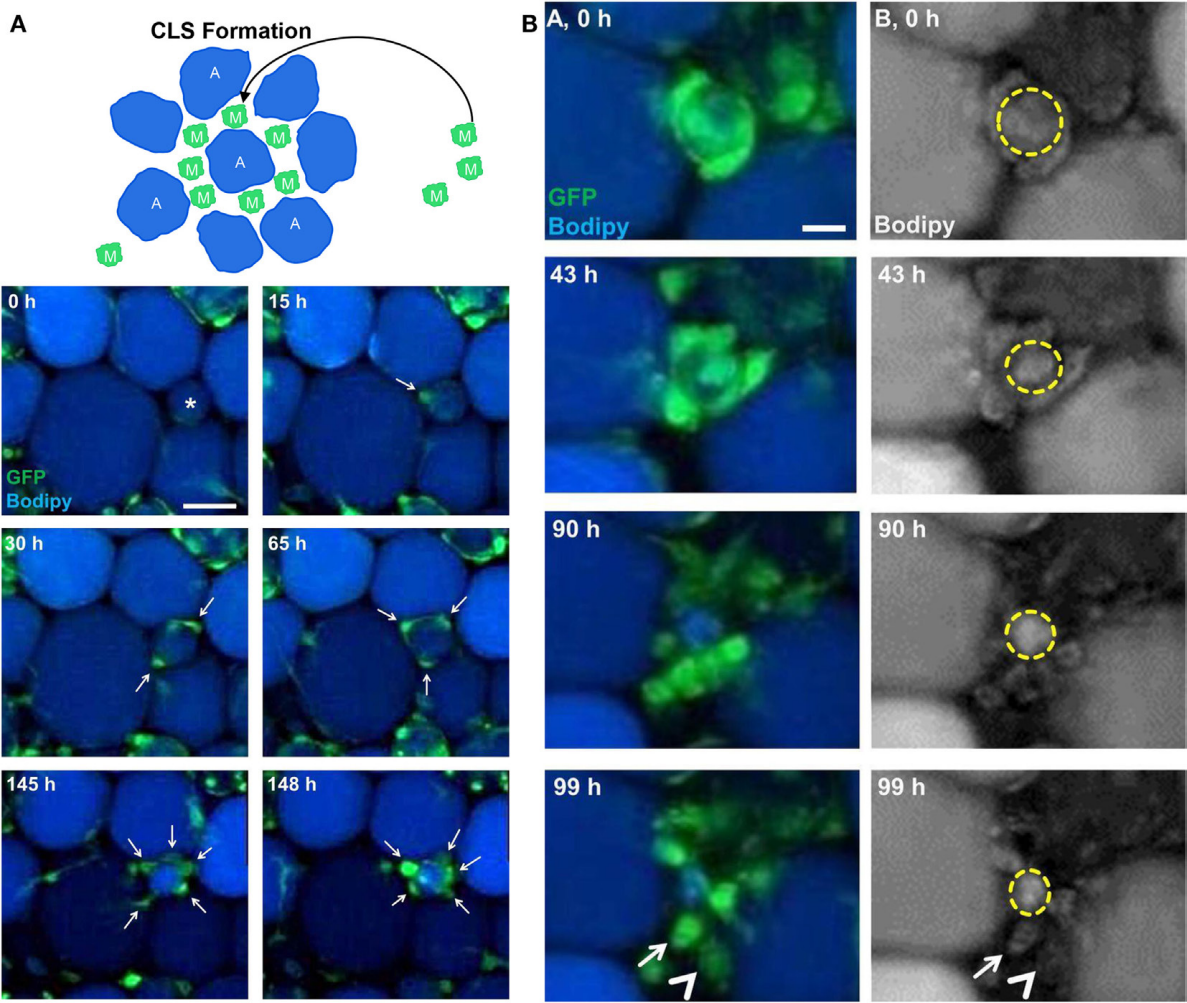
Imaging macrophages in the context of obesity. **(A)** Long-term imaging showed that enhanced green fluorescent protein (EGFP)-expressing macrophages (*M*) migrated toward and accumulated around dying adipocytes (*A*) stained with BODIPY to form crown-like structure (CLS) in *ex vivo* cultured adipose tissue explants from a mouse (reproduced under the terms of the CC BY 4.0 license, Copyright© 2015 the American Physiological Society) (92). **(B)** EGFP-expressing macrophages were involved in degradation of dead adipocytes in adipose tissue explants from a mouse (reproduced under the terms of the CC BY 4.0 license, Copyright© 2015 the American Physiological Society) (92).

Adipocyte hypertrophy and cellular stress lead to adipocyte death, which is a common pathogenesis mechanism for obesity-associated inflammation in adipose tissue. Under lean conditions, adipocytes secrete anti-inflammatory factors to stimulate M2 activation of ATMs. In return, M2 macrophages produce anti-inflammatory factors to inhibit M1 activation of ATMs, maintaining adipose tissue homeostasis. Under obesity conditions, abnormal adipocytes secret pro-inflammatory factors, such as free fatty acids and TNF-α, to promote M1 activation of ATMs and to recruit more monocytes (7). Recently, Gericke et al. developed a live imaging method that allowed for more than 7 days of tracking of macrophage behavior in cultured adipose tissue explants from CSF1R-EGFP mice. Long-term imaging showed that EGFP-tagged macrophages migrated toward and accumulated around dying adipocytes (stained with BODIPY dye) to form a CLS (Figure 4A). Subsequently, macrophages in the CLS engulfed the lipid remnants of perishing adipocytes (Figure 4B) (92). These behaviors of ATMs under inflammation conditions are in line with previous studies. As a response, activated M1 macrophages secrete various pro-inflammatory cytokines, which act on adipocytes, and chemokines to recruit more macrophages. Sekimoto et al. revealed the interaction between macrophages and adipocytes using intravital imaging. *In vivo* live imaging showed that EGFP-expressing macrophages became activated to migrate just 5 days after obesity stimulus in LysM-EGFP mice. Adipocyte-derived S100A8 protein had the capability to stimulate chemotactic migration of EGFP-labeled ATMs *in vivo* and RAW 264.7 cells *in vitro*. This study showed that adipocyte-derived cytokines triggered the early migration of ATMs, which eventually resulted in progression of chronic inflammation in adipose tissue (93). In other words, adipocytes are the initiator of the inflammatory response, whereas ATMs are critical mediators to propagate inflammation in adipose tissue.

Aside from chronic local inflammation, dysfunction of lipolysis is also a cause leading to obesity. Under homeostasis, leptin, a hormone secreted from adipose tissue, mainly acts on the brain and then stimulates the lipolytic effect through the action of sympathetic nerve fibers in adipose tissue. Norepinephrine is a circulating hormone produced by the ends of sympathetic nerve fibers and facilitates the association between sympathetic neurons and adipose tissue (154). Recently, Pirzgalska et al. identified a new population of sympathetic neuron-associated macrophages (SAMs) related to obesity in mice by using live imaging. Intravital multiphoton imaging depicted that GFP-tagged macrophages exhibited differentiated morphology from ATMs for specific association with sympathetic nerve fibers in the LipidTOX-labeled fat pad of mice. Transcriptomic analysis showed that SAMs highly expressed the genes encoding neurotransmitter receptors, transporters, and catalytic enzymes. This study indicated that SAMs contributed to obesity by possessing the machinery for uptake and degradation of norepinephrine (94).

In conclusion, a positive feedback loop of paracrine between ATMs and adipocytes propagates inflammation and further decreases insulin sensitivity in adipose tissue (2, 7). Furthermore, obesity is a risk factor for certain kinds of cancer and type 2 diabetes mellitus (155). Obesity-related complications are frequently diagnosed late in the course of progressing disease. Thus, the identification and analysis of macrophages in adipose tissue may be a potential way to prevent, predict, or even reverse the cardiovascular and metabolic complications.

### Atherosclerosis

Atherosclerosis is a chronic inflammatory disease triggered by lipid retention in the arterial wall and is characterized by vessel wall thickening and lumen narrowing (156). Atherosclerotic lesions, also called plaques, persist on the inside of arteries, such as the coronary artery, cerebral artery, and renal artery. Murine aortas are the most commonly used tissues for pathological examination in preclinical atherosclerotic models. Therefore, the penetration depth is the main technical limitation for optical imaging techniques to track macrophage dynamics in the context of atherosclerosis.

The intravascular imaging system has been applied to examine atherosclerosis by implanting a catheter. For example, Jaffer et al. developed a near-infrared fluorescence (NIRF) catheter-based strategy to image cysteine protease activity during vascular catheterization, which provided a platform to detect the inflammation of atherosclerotic vessels in a rabbit model (96). Recently, Kim et al. constructed an injectable NIRF probe to image macrophages in high-risk plaques by targeting macrophage mannose receptors. They directly visualized the plaque macrophages in murine carotid atheroma by using a custom-built IVM under the surgical exposure of carotid arteries. Moreover, they also imaged the plaque inflammation of atheromatous vessels in rabbits by using a catheter-based imaging system (95). To date, the application of catheter guided imaging is mainly limited to large animal models, such as rabbits, due to technical restrictions. However, other imaging techniques have been successfully used to diagnosis atherosclerosis in the clinic, such as X-ray examination, PET, Doppler ultrasonography, optical coherence tomography, intravascular ultrasound, and NIR spectroscopy (157, 158). These imaging techniques are also used to study atherosclerosis pathogenesis in basic and preclinical research, which has been well reviewed elsewhere (159–161). For instance, PET has been widely used to track inflammation in atherosclerosis due to its deeper penetration ability and higher sensitivity. Nahrendorf et al. developed a novel trimodality nanoparticle to detect macrophages in atherosclerotic plaques *in vivo*. After injection of the PET tracer ^64^Cu, PET-CT imaging showed that a strong PET signal was detected in vascular regions with a high plaque burden in an atherosclerotic mouse, whereas no activity was observed in the same vasculature of wild-type mice. This study indicated that PET had the capability to spatially localize the atherosclerotic plaques in preclinical practice (19). Ye et al. explored fluorine-18 fluorothymidine (^18^F-FLT) PET-CT imaging to examine cell proliferation in atherosclerotic plaques of mice, rabbits, and human patients. ^18^F-FLT (^18^F-fluorothymidine), a thymidine analog, avidly enriches in proliferating cells. PET-CT images showed higher ^18^F-FLT uptake in atherosclerotic lesions in an atherosclerotic mouse compared with a wild-type mouse. Moreover, *in vivo* PET-CT imaging after fluorouracil (5-FU, a cell proliferation inhibitor) injection showed that 5-FU treatment reduced ^18^F-FLT uptake in mouse atherosclerotic plaque. Immunofluorescence staining indicated that the majority of proliferating cells in atherosclerotic lesions were macrophages, and local cell proliferation may be a source for plaque macrophages. This study indicated that ^18^F-FLT PET imaging can serve as an imaging biomarker for cell proliferation in plaques and gave us the idea for imaging cell proliferation *in vivo* (37).

With the help of imaging techniques and histological methods, accumulating evidence supports the idea that macrophages play a decisive role at all stages of atherosclerosis progression (162). M1 macrophages are implicated in plaque initiation and progression, while M2 macrophages participate in plaque stabilization (6). In the early phases of atherogenesis, low-density lipoprotein (LDL) is abnormally accumulated in intima where LDL is more susceptible to oxidation. Oxidized LDL particles trigger an inflammatory reaction in endothelial cells; they then secrete mediators into the blood circulation to recruit circulating monocytes. The monocytes transmigrate across arterial walls and differentiate into macrophages in the intima, where they are transformed into foam cells by the bulk oxidized LDL. Foam cells are the foundation for atherosclerotic plaque formation. Foam cells secrete cytokines to propagate inflammatory responses and attract more monocytes by producing chemokines. However, M2 macrophages were also detected in the atherosclerotic plaques (163). It has been demonstrated that M2 macrophages were present in more stable regions of plaques and were more resistant to foam cell formation (164). Therefore, the presence of M1 and M2 macrophages may reflect the plaque progression or regression. Given the importance and various macrophage functions in atherosclerosis, future studies should focus on the development of macrophage-targeting therapies.

## Limitation and Perspectives

Bioluminescence imaging has many advantages, such as having no need for light excitation, being background free, being non-invasive, having high throughput capability (five mice) and deep penetration ability, and being able to support long-term intermittent tracking. Advanced BLI has been widely used to monitor tumor growth, detect metastasis, and evaluate the responses to therapies in preclinical practice. There are some limitations for BLI regarding the observation of macrophages *in vivo*, such as poor spatial resolution and short tracking duration (24). However, there are many solutions to these problems. First, the advanced *in vivo* imaging system (IVIS) equipped with tomographic technologies has the capability to import and automatically co-register CT or MRI images, which greatly improves the anatomical resolution of BLI. Second, anesthesia is a factor limiting the tracking duration of BLI. It is still a technical challenge to avoid excessive anesthesia for mice in a larger imaging chamber. Having individuals inhale anesthesia by a mask or breathing tube may be a feasible way to conduct personalized anesthesia. Last, chemiluminescence is another factor leading to the short tracking duration of BLI *in vivo*. Recently, GFP as an alternative probe has been used to resolve the short duration problem, but GFP-transfected cells are limited to superficial tissues (29). Moreover, advanced IVIS is equipped with a full range of excitation and emission filters, a feature that allows for the use of a great variety of NIRF probes. Various macrophage-targeting NIRF nanoprobes have been used to conduct real-time monitoring of inflammatory responses *in vivo* (165–168). Therefore, IVIS is still a preferable platform for whole-body imaging, which is suitable for tracking macrophage recruitment, evaluating the content of macrophages in tissue, and tracing macrophage elimination.

Compared with other imaging techniques, IVM has many unique advantages, such as having low invasiveness, microscopic resolution, real-time imaging, and long-term tracking capabilities. With the development of novel nanoprobes and transgenic-labeled reporters, IVM provides an established platform to track and analyze macrophage dynamics over a long duration in native microenvironment *in vivo*, such as cell motility, cell–cell interactions, and physiological behaviors. However, there are some technical limitations for IVM, such as poor penetration depth, poor animal survival under long-term anesthesia, and imprecise cell labeling (Table 3). The penetration depth is an important technical limitation for IVM; thus, IVM imaging is limited to relatively superficial tissues. This will impede studies on the macrophages of internal organs, such as the liver and lung. Some strategies have been developed to overcome the limited penetration depth of IVM, such as surgical exposure, installation of optically transparent windows, and the use of endoscopy (41, 169–171). Optical windows have become an indispensable technique to obtain microscopic access to various organs *in vivo*. The critical steps for optical window usage are implantation and fixation. For example, the dorsal skinfold chamber is fixed on the skin using screw spike (172), the cranial window is secured on the skull using glue after skin exposure and bone incision (173), the mammary imaging window is sandwiched between the mammary gland and the skin using a surgical suture after skin incision (174), the abdominal imaging window is fixed on the skin–muscle layer using surgical suture after skin–muscle layer incision (170), and the thoracic suction window is fixed on the intercostal ribs after skin-parietal pleura layer incision (175, 176). The first three imaging windows tightly stick to the target organs under the mechanical pressure produced during window implantation. However, the organs of interest in the chest or abdominal cavity may deviate from optical windows due to internal traction forces or anatomical positions, especially when the animals are mounted in the supine position on the stage of an inverted microscope. Inner negative pressure *via* air exhaust has been used to suck the tissues and shorten the distance between the optical window and organs (175, 176). Last, physiology is inevitably disturbed by using these approaches (especially surgery and window implantation), giving rise to inflammation and fluid leakage; therefore, it is urgent to develop a non-invasive method for resolving penetration depth.

**Table 3:** Challenges and solutions for optical imaging techniques during *in vivo* macrophage tracking.

Platforms	Limitations	Solutions and perspectives	Reference
Bioluminescence imaging	Short duration	Green fluorescent protein or near-infrared nanoprobes	(29, 165–168)
	Poor spatial resolution	Combination with tomography or intravital microscopy (IVM)	
	Excessive anesthesia	Individual inhale and real-time monitoring	

IVM	Poor penetration depth	Surgical exposure	(169)
		Optically transparent window	(170)
		Endoscopy	(171)
		Multiphoton microscopy	(177)
	Long-term anesthesia	Humidified anesthetic gas	(178)
		Real-time monitoring of vital signs	(179)
		Heating pad to avoid hypothermia	(169)
		Saline injection to compensate fluid loss	(178)
	Imprecise cell labeling	Multiple labeling	(79)
		Multicolor labeling or Brainbow technology	(82, 180)
		Third harmonic generation microscopy or fluorescence lifetime imaging microscopy	(49, 60, 61, 181)

Clearly, multiple labeling is the primary technique for *in vivo* observation of individual cells in their native multicellular microenvironment. However, the application of multiple labeling in macrophage studies falls into a dilemma. First, the most commonly used fluorescent proteins have overlapping emission spectra (Figure 1B). A routine approach is to sacrifice high signal intensity to ensure that there is no cross-channel contamination. Second, although there are many transgenic methods to label myeloid cells (macrophages and other phagocytes), currently, there are a limited number of non-invasive methods available to distinguish macrophages from neutrophils (48). Brainbow technology is a classic case used to distinguish single neurons from neighboring neurons in the brain using random expression of multiple fluorescent proteins (182). With the development of gene editing technology, such as the Cre–Lox system and the CRISPR–Cas9 system, next generation Brainbow variants have been successfully used to trace the lineage of multiple progenitors and to track the development of the corneal epithelium in model organisms (180, 183). It is clear that the Brainbow technique will become a potential tool for the future to carry out fate mapping of myeloid cells and to visualize the phenotype of polarized macrophages.

By exploiting the endocytosis activity, macrophages can also be labeled using fluorophore–dextran conjugates and quantum dots. Quantum dots have a relatively narrow emission bandwidth (10–20 nm), and the emission peak wavelength depends on their size. This unique optical property of quantum dots can reduce cross-channel contamination in multiple-labeling imaging (184). However, it is difficult to distinguish macrophages from neutrophils when using exogenous fluorescent labeling based on endocytosis activity. Furthermore, cytotoxicity is also a particular concern and needs to be addressed before preclinical studies and clinical translations (185). Cellular uptake is the main cause for cytotoxicity. Although macrophages are the main phagocytes in the body, other cells also have the ability to uptake the nanoprobes, such as dendritic cells, monocytes, neutrophils, NK cells, and endothelial cells (38). Modification of the size and surface of nanoprobes is an effective way to achieve the selective uptake of targeted cells. First, conjugation of peptides or ligands on the surface of probes improves the targeted delivery of nanoprobes. For instance, antibodies (anti-CD206 antibody, macrophage mannose receptor) and ligands (mannose, folate) have been used to conduct macrophage imaging *in vivo* (186, 187). Second, the particle size affects the organ distribution of nanoprobes. Generally, large nanomaterials (>1,000 nm in diameter) are stuck in the capillaries of the liver and lungs, medium-sized nanomaterials (10–300 nm in diameter) typically accumulate in the organs containing high numbers of macrophages (such as the liver, the spleen, lymph nodes, and disease tissues), and small nanoparticles (<8 nm in diameter) are mainly cleared through the kidneys (38). Last, the surface properties (charge and its density) also affect the pharmacokinetics and organ distribution of nanoprobes. Generally, positively charged nanoprobes preferentially translocate from the bloodstream to targeted tissues and enter the cells because the lumen layer of vessels and the outer membrane of cells are negatively charged. Although positive charge appears to improve the efficacy of cellular uptake, a higher cytotoxicity of such nanoprobes has been reported (188). Therefore, tailor-made construction of nanoprobes is required to achieve the precision labeling of different macrophage populations.

Although multiple labeling is a potential method for labeling macrophages, specifically *in vivo*, by employing GFP and fluorescent dye double labeling (79), label-free imaging techniques are still the preferred tools to achieve the least perturbation to the native microenvironment by reducing labeling requirements. THG microscopy will be an alternative choice of modality that can provide complementary information in the immune microenvironment. Recently, our group found that *in vivo* THG microscopy has the capability to identify different leukocytes based on their granularity differences in mice under LPS challenge (60). Moreover, fluorescence lifetime imaging microscopy (FLIM) may become an alternative technique for imaging macrophages *in vivo* without the use of labeling. Metabolic reprogramming occurs concurrently with the polarization of macrophages (189). Recently, Alfonso-García and colleague employed the metabolism shift as an endogenous marker to identify macrophage phenotypes using FLIM. This study indicated that FLIM was a non-invasive platform to identify different subtypes of macrophages (181). Szulczewski et al. developed a novel imaging method to carry out *in vivo* visualization of stromal macrophages in tumors. This method exploited the remarkable difference in the intensity and fluorescence lifetime of metabolic cofactors (NADH and FAD) between tumor cells and stromal macrophages *via* IVM and label-free FLIM, respectively (49).

The ideal situation is to observe immune cells in detail without disturbing them. Unfortunately, previous studies indicate that some common injectable anesthetics have the potential for adverse effects on lymphocyte trafficking (190, 191). Recently, gas (isofluorane) anesthesia has become an alternative approach for small animal anesthesia, and gas anesthesia is easier to maintain over long-term intervals. However, animal care under anesthesia is also a crucial problem during long-term imaging. Some possible solutions have been suggested to improve long-term anesthesia-related survival, such as humidified gas anesthesia, real-time monitoring of vital signs, the use of a heating pad to keep the body of the mouse warm, and saline injection to compensate for fluid loss (169, 178, 179). There are multiple factors controlling animal survival during anesthesia, including anesthesia equipment, operator proficiency and animal physiological status. Thus, long-term anesthesia is difficult to reproduce in different research institutes, and it is necessary to develop a standard operating procedure of anesthesia if scientists want to conduct long-term imaging of macrophages in animals.

## Conclusion

In this article, we have mainly focused on reviewing the advances in optical imaging techniques (BLI and IVM) for discovering the roles of macrophages during disease progression. Although optical imaging techniques greatly advance the development of macrophage biology, various interesting phenomena from traditional experiments still need to be analyzed and visualized *in vivo*. For instance, the MMT is a debated topic in the field of renal fibrosis (142, 143). Intravital imaging using SHG and THG may provide more visual details of the MMT in the future. Moreover, precise labeling, long-term tracking and the penetration depth are major challenges for the optical imaging of macrophages. Taken together, with continuing progress in multidisciplinary cooperation, such as optical engineering, nanophotonics, and anesthetization technology, we expect that optical imaging techniques will continue to provide valuable platforms to investigate macrophage biology in different tissues and disease contexts.

## Nomenclature

**Table:** 

ATMs	adipose tissue macrophages
BLI	bioluminescence imaging
CFP	cyan fluorescent protein
CLS	crown-like structure
CT	computed tomography
EGFP	enhanced green fluorescent protein
ECFP	enhanced cyan fluorescent protein
ECM	extracellular matrix
effluc	enhanced firefly luciferase
FLIM	fluorescence lifetime imaging microscopy
GFP	green fluorescent protein
GMPs	granulocyte/macrophage progenitors
IVIS	*in vivo* imaging system
IVM	intravital microscopy
IL-4	interleukin 4
IFN-γ	interferon-γ
JAM-C	junctional adhesion molecule-C
LDL	low-density lipoprotein
LPS	lipopolysaccharide
MRI	magnetic resonance imaging
MMP	metal matrix proteinase
MMT	macrophage-to-myofibroblast transition
NIR	near-infrared
NIRF	near-infrared fluorescence
PET	positron emission tomography
PET-CT	positron emission tomography-computed tomography
RFP	red fluorescent protein
SAMs	sympathetic neuron-associated macrophages
SHG	second harmonic generation
SPECT	single-photon emission computed tomography
TAMs	tumor-associated macrophages
TDA	tumor-derived antigen
THG	third harmonic generation
TMEM	tumor microenvironment of metastasis
TMR	tetramethylrhodamine
TNF	tumor necrosis factor
YFP	yellow fluorescent protein
3D	three-dimensional
4D	four-dimensional
5-FU	fluorouracil
^18^F-FDG	fluorine-18 fluorodeoxyglucose
^18^F-FLT	fluorine-18 fluorothymidine

## Author Contributions

All authors listed have made a substantial, direct, and intellectual contribution to the work and approved it for publication.

## Conflict of Interest Statement

The authors declare that the research was conducted in the absence of any commercial or financial relationships that could be construed as a potential conflict of interest.
